# Elongated Uvula

**Published:** 1892-05-21

**Authors:** 


### THE PRACTITIONER'S MIRROR AND RETROSPECT.

[*The Editor will be glad to receive offers of co-operation and contributions from members of the profession. All letters should be addressed to* The Editor, The Lodge, Porchester Square, London, W.]

### MEDICINE.

### ELONGATED UVULA.

The symptoms which an elongated uvula may originate are so very pronounced that it would probably be difficult for any practitioner, who was not acquainted with the "modus operandi" of such an apparently slight deviation from a natural condition, to realise the possibility of their source.

Labus of Milan very conveniently classifies cases of elongated uvula under two heads, viz., (1) those in which the uvula and soft palate are merely relaxed, the general appearance of the parts remaining practically normal. In these there is no marked congestion or infiltration of the parts, although a slightly increased vascularity may be noticeable. The mucous membrane covering the muscles of the uvula and velum palati is somewhat redundant and is seen to extend beyond the limits of the muscular velum, and to form a semi-translucent portion projecting down from the tip of the uvula. (2) Those in which there is a very considerable degree of hypertrophy and inflammatory infiltration, more especially of the uvula, rendering it much thicker and obviously longer than it should be. The vessels of the parts are markedly full and congested, and the fauces generally participate in the condition of degeneration that renders the mucous membrane thickened and irregular on the surface. In this second group of cases a varicose condition of the veins of the base of the tongue is frequently observed.

The slighter cases often result from a general want of tone, from oft recurring nasal catarrh, or from using the voice unduly, or by a wrong method of singing or speaking. They may all pass into the more aggravated condition of elongation with hypertrophy and chronic pharyngitis. But the more pronounced cases are generally brought about from the pharyngeal congestion which results from portal congestion in cirrhosis of the liver, or from venous stagnation in mitral regurgitation. Not infrequently chronic constipation and dyspepsia by inducing a congestion of the pharynx in which the soft palate participates becomes the source of all the trouble. One must bear in mind that nasal disease, such as chronic hypertrophic rhinitis, or the presence of a polypus may also cause the condition from the resulting paresis of the soft palate. It is common, too, in phthisis pulmonalis, or in any disease with continual coughing. The symptoms of elongated uvula are by no means proportionate to the condition, and it is surprising how an apparently slight elongation will bring about all the worst symptoms, while, on the other hand, the most obviously congested and much elongated uvula gives rise to no symptoms at all.

In a fairly typical case the patient complains of tickling at the back of the throat, often described as a sensation as though there was a hair in the larynx that could not be got rid of. The continual irritation of the tip of the uvula against the dorsum of the tongue, and the constant coughing and hawking it induces only aggravates the initial mischief. It is chiefly at night in lying down that the tickling is so disturbing, and in fact the faucial irritation is so great as to make the patient vomit. Vomiting, however, may occur after every meal. Lennox Browne mentions the case of a medical man who consulted him, "complaining of constant pain in the left subscapular region, with irritable cough, loss of flesh, and impairment of general health. On the recommendation of two physicians, eminent in chest diseases, he sold his practice; but he entirely recovered his health after removal of his uvula, and fourteen years later was still active, and engaged in professional work." It is not surprising that the loss of sleep and frequent vomiting should result in great emaciation and weakness, which, in association with cough, with expectoration of mucus streaked with blood from the pharynx, should lead one to suspect that the patient is suffering from tubercular disease of the lungs, especially in those who also complain of localised pains in the chest—pains which are purely reflex in origin. Consequently, whenever patients are suspected to be phthisical on account of their general aspect and condition, but in whom the absence of abnormal physical signs in the lungs seem sufficient to contravene the diagnosis it is well to bear in mind the possibility of elongated uvula. Another urgent symptom that may occur is obstructive dyspnœa. We have seen a patient who suffered from severe and urgent attacks of dyspnœa on lying down at night, in addition to other minor symptoms, and thought to be due to some serious laryngeal affection. The dyspnœic attacks were the result of spasm of the glottis, caused by an elongated uvula; removal of the uvula prevented a recurrence of these alarming attacks.

There is, of course, no difficulty whatever in diagnosing the conditions included under Labus' second head, but when the uvula is simply elongated it may not be very obvious at first sight. The patient should be directed to keep his mouth open and strike a high pitched note while the tongue is depressed with a spatula. The mucous membrane is thrown into unnatural transverse folds instead of being withdrawn into the retracting soft palate, and the elongated portion remains hanging down.

The treatment is simple. Any tendency to constipation should be met by the administration of saline purgatives. This, with a suitable tonic, and painting the soft palate with a solution of perchloride of iron (60 grs. of the anhydrous salt to the ounce of water), or some other suitable astringent may relieve the milder cases. Generally, however, it becomes necessary to remove the elongated mucous membrane by seizing it with a pair of forceps and gently pulling it downward and forwards to snip it off with curved scissors. The employment of cocaine renders the simple procedure almost painless.

